# Psychological Dimensions of Hair Transplantation: A Narrative Review of Current Evidence

**DOI:** 10.1111/jocd.70475

**Published:** 2025-09-24

**Authors:** Isabella J. Tan, Mohammad Jafferany

**Affiliations:** ^1^ Robert Wood Johnson Medical School, Rutgers The State University of New Jersey Piscataway New Jersey USA; ^2^ Department of Psychiatry and Behavioral Sciences Central Michigan University College of Medicine Mount Pleasant Michigan USA

**Keywords:** alopecia, body dysmorphic disorder, hair transplantation, mental health, patient expectations, psychological impact

## Abstract

**Background:**

Hair transplantation is a widely performed intervention for alopecia, offering both cosmetic restoration and psychological benefits. Increasing evidence highlights the psychosocial impact of hair loss, particularly regarding self‐esteem, identity, and social functioning.

**Aims:**

This review aims to evaluate the psychological dimensions of hair transplantation, including patient motivations, emotional burden, the role of psychological screening, and the associated mental health outcomes.

**Methods:**

A structured narrative review was conducted using PubMed and Scopus databases to identify peer‐reviewed articles published up to May 31, 2025. Search terms included “hair transplantation,” “alopecia,” “psychological impact,” “body dysmorphic disorder,” “depression,” and “quality of life.” Studies focusing on adult patients undergoing hair transplantation and reporting psychological outcomes or screening practices were included.

**Results:**

Hair loss is associated with significant psychological distress and may exacerbate depression, anxiety, and social withdrawal. Screening tools such as the Body Dysmorphic Disorder Questionnaire (BDDQ) and Beck Depression Inventory (BDI) are effective in identifying high‐risk individuals. When patient expectations are well managed and psychological risk factors are considered, hair transplantation can lead to improved self‐esteem, confidence, and emotional well‐being. Conversely, inadequate screening or poor patient selection may result in dissatisfaction or worsening mental health.

**Conclusions:**

Incorporating psychological evaluation into preoperative assessment must be considered for optimizing surgical and mental health outcomes. A multidisciplinary approach involving dermatologists, surgeons, and mental health professionals is recommended. Further research should establish standardized guidelines for psychosocial screening in hair restoration.

## 1 Introduction

Hair transplantation has evolved into a widely practiced and increasingly sophisticated cosmetic procedure, offering individuals with alopecia an opportunity to regain not only hair but also a sense of normalcy, identity, and psychological well‐being. Although often categorized as an *esthetic* intervention, its impact extends far beyond physical appearance [1]. For many patients, hair loss is not merely a cosmetic issue—it is a deeply personal experience that can disrupt self‐image, reduce self‐esteem, and impair social functioning [1]. The visible nature of scalp alopecia, especially in sociocultural contexts where hair is strongly associated with youth, vitality, and attractiveness, can precipitate significant emotional distress. Studies have shown that individuals with hair loss frequently report feelings of embarrassment, shame, and diminished self‐confidence, sometimes leading to anxiety, depression, and social withdrawal [2].

The psychological burden of alopecia is particularly pronounced in certain populations, including women and younger individuals, for whom societal pressures and gender norms may intensify the emotional impact [3]. Despite the prevalence of these psychosocial effects, the psychological dimensions of hair transplantation remain underexamined in both clinical practice and literature. While surgical outcomes and graft survival have historically dominated research in this field, there is growing recognition that patient‐reported outcomes and psychological metrics are equally critical indicators of success.

This review synthesizes current evidence on the psychological aspects of hair transplantation, emphasizing a patient‐centered framework that spans the entire treatment—from preoperative consultation and psychological screening to postoperative satisfaction and long‐term psychosocial adjustment. Focus areas are the emotional sequelae of hair loss, the spectrum of patient motivations (ranging from reconstructive needs to body image enhancement), and the utility of validated psychological assessment tools such as the Body Dysmorphic Disorder Questionnaire (BDDQ) and the Beck Depression Inventory (BDI). Additionally, the review discusses risk factors for postoperative dissatisfaction, including unrealistic expectations and underlying psychiatric conditions, which can compromise outcomes despite technically successful procedures. This review advocates for a multidisciplinary, ethically grounded approach to hair transplantation, integrating the expertise of dermatologists, hair restoration surgeons, and mental health professionals. By addressing both the physical and psychological needs of patients, clinicians can enhance the therapeutic potential of hair restoration and ensure that surgical success translates into meaningful, durable improvements in quality of life.

## 2 Materials and Methods

A structured narrative review was conducted using PubMed and Scopus databases to identify peer‐reviewed articles published up to May 31, 2025. Search terms included “hair transplantation,” “alopecia,” “psychological impact,” “body dysmorphic disorder,” “depression,” “anxiety,” and “quality of life,” combined using Boolean operators. Studies were eligible if they involved adult patients (≥ 18 years), were published in English, and addressed psychological outcomes of hair transplantation or the use of psychological screening tools such as the BDDQ or BDI. Exclusion criteria included studies focused solely on surgical techniques, pediatric populations, non‐peer‐reviewed content, and articles not available in English. Titles and abstracts were screened by two independent reviewers, followed by full‐text review for eligible studies. Disagreements were resolved through consensus or consultation with a third reviewer. Extracted data included study design, sample size, patient characteristics, psychological variables assessed, screening tools used, and main findings. Risk of bias in individual studies was assessed using the Joanna Briggs Institute Critical Appraisal Checklists appropriate for study design (e.g., cross‐sectional, cohort, or case series), with each study evaluated independently by two reviewers and discrepancies resolved by discussion. Due to heterogeneity in study design and outcomes, a narrative synthesis approach was used in lieu of formal systematic review and meta‐analysis.

## 3 Results

Hair holds profound significance in personal identity, cultural norms, and perceptions of attractiveness. Consequently, hair loss can lead to distress that extends well beyond cosmetic concern [3]. When hair thinning begins prematurely, progresses rapidly, or results in visible scalp exposure, individuals may experience a crisis of self‐perception, impaired social functioning, and diminished psychological well‐being. For many, hair symbolizes health, youth, and gender expression: full hair in men often connotes masculinity, while in women it reflects beauty and femininity [4]. These meanings are reinforced by media, culture, and personal belief systems, which is why even early‐stage alopecia can trigger disproportionate emotional distress. Patients frequently report a loss of control over their appearance and aging process, with associated concerns about their professional image, social status, and romantic desirability [4].

The psychological burden of alopecia has been widely documented across subtypes, particularly androgenetic alopecia (AGA), one of the most studied forms [5]. A cross‐sectional descriptive study found that patients with AGA commonly experience reduced self‐esteem, heightened self‐consciousness, and a perceived loss of attractiveness [5], with 48.33% reporting significant self‐consciousness and a mean dermatology life quality index (DLQI) score of 2.79 [5]. Many adopt avoidance behaviors, such as avoiding photos, mirrors, or social events—especially as scalp visibility increases [6]. These reactions are compounded by the chronic nature of hair loss and its perceived stigma. Cultural and gendered differences also play a role: while some societies view male baldness as a marker of wisdom or maturity, others see it as a loss of virility and appeal [7]. For women, hair loss can carry strong psychosocial implications due to associations with femininity; however, men are also deeply affected, though they may be less likely to express or report concerns [8].

Validated tools such as the DLQI and Hairdex Quality of Life Instrument quantify the psychosocial impact of hair loss, revealing a quality‐of‐life burden comparable to or exceeding that of chronic dermatologic conditions like psoriasis and eczema [6, 9]. Hair loss may not only provoke emotional discomfort but also serve as a trigger or aggravating factor for psychiatric conditions [10]. In some cases, it may initiate clinically significant anxiety or depression; in others, it exacerbates preexisting mood disorders [6]. A subset of patients also exhibits distorted perceptions of their hair loss, sometimes indicative of body dysmorphic disorder (BDD) [11]. These individuals may obsess over minimal or imagined defects, seek repeated interventions, and develop unrealistic expectations for surgical results [11].

Expectation management is a critical determinant of patient satisfaction in hair restoration [12]. Since hair carries symbolic and emotional weight, some individuals arrive with unrealistic expectations—believing the procedure will fully restore lost youth, resolve self‐esteem issues, or dramatically change their social lives [13]. Misinformation from social media, advertising, or anecdotal reports often fuels these beliefs. If not corrected, such expectations increase the risk of dissatisfaction, emotional distress, or even conflict and litigation postoperatively [14]. Additionally, dissatisfaction can arise from consultations that are inadequate, incomplete, or potentially misleading, particularly when provided by individuals who are not licensed medical professionals [14]. Such consultations may overpromise results, underestimate limitations, or fail to adequately assess psychological readiness, thereby increasing the risk of postoperative disappointment and emotional distress.

Structured psychological tools including the BDDQ, BDI, and Generalized Anxiety Disorder Scale (GAD‐7) are useful in identifying patients who may be at risk of poor outcomes due to mood disorders, anxiety, or distorted beliefs (Table 1) [16, 17]. Positive screening does not disqualify a patient but signals the need for further psychiatric assessment.

**TABLE 1:** Recommended psychological screening tools for hair transplantation candidates.

Tool	Domain assessed	Format/Cutoff	Clinical purpose	Significance in hair transplantation
Body Dysmorphic Disorder Questionnaire (BDDQ)	Body dysmorphic disorder (BDD)	Single‐page self‐report; positive screen if preoccupation causes significant distress/impairment	Flags patients who may require further psychological evaluation	Identifies individuals at risk of unrealistic expectations or persistent dissatisfaction; helps prevent poor surgical outcomes and ethical dilemmas [15]
Patient Health Questionnaire‐9 (PHQ‐9)	Depression severity	9 items; score ≥ 10 = moderate depression	Quick assessment of depressive symptoms	Detects mood disturbances that could affect recovery, expectation management, or postoperative satisfaction [16]
Generalized Anxiety Disorder‐7 (GAD‐7)	Anxiety severity	7 items; score ≥ 10 = moderate anxiety	Screens for clinically relevant anxiety	Identifies patients whose anxiety may interfere with preoperative counseling, adherence, or postoperative coping [16]
Hospital Anxiety and Depression Scale (HADS)	Anxiety & depression	14 items; subscale ≥ 8 = possible case	Screens for both anxiety and depression simultaneously	Useful for patients with mixed mood symptoms; validated in dermatology and cosmetic surgery populations [17]

When properly indicated and conducted, hair transplantation can yield significant psychological benefits. Patients often report improvements in self‐esteem, body image, and social confidence [4]. Surveys show high satisfaction rates—ranging from 75% to 90%—particularly among those with realistic expectations [18]. While hair transplantation generally improves psychological well‐being, current evidence suggests minimal differences in mental health outcomes between follicular unit extraction (FUE) and follicular unit transplantation (FUT) methods; patient satisfaction appears more closely linked to expectation management and overall esthetic result than to the specific surgical technique used [2].

Hair transplantation can also act as a catalyst for broader psychological growth. Patients who regain a sense of agency in their appearance may experience increased self‐care, motivation, and emotional resilience. Nonetheless, psychological risks cannot be overlooked. Postoperative dissatisfaction may occur due to complications or misaligned expectations [14]. In particular, patients with BDD may fixate on minor imperfections and remain persistently dissatisfied despite favorable results [16]. Because BDD is considered a contraindication for elective cosmetic procedures, early screening with validated tools like the BDDQ is critical [15]. Other risks arise during the regrowth phase, when visible improvements take months [2]. Temporary feelings of doubt, impatience, or regret are common, and in vulnerable individuals, these may progress to clinical symptoms [19]. Surgical complications—such as graft failure, unnatural esthetic outcomes, or visible scarring—can further amplify distress [20, 21]. Severe cases may lead to emotional crises, including loss of self‐worth or depressive episodes, requiring both surgical and psychological intervention [22].

## 4 Discussion

The meaning of hair and the psychosocial impact of hair loss are influenced by cultural norms, gender expectations, and personal beliefs [3, 4]. Emotional distress associated with alopecia often involves a loss of control over appearance, professional image, social standing, and romantic desirability [4]. Understanding these psychosocial dynamics is critical for optimizing patient outcomes in hair restoration.

Psychological screening—using validated instruments such as the BDDQ, BDI, and GAD‐7—helps identify patients at risk for poor outcomes due to mood disorders, anxiety, or distorted beliefs [16, 17]. Positive screening does not disqualify patients but signals the need for psychiatric assessment before surgery. Surgeons must act as educators, communicators, and gatekeepers, using preoperative counseling to clarify biological limitations, the gradual timeline of regrowth, and variability in individual response [23]. Visual aids, such as digital simulations or before‐and‐after photos, can help anchor expectations, but practitioners must exercise caution: simulations may depict idealized outcomes that cannot always be achieved in reality, and presenting them carries potential legal liability [23].

Patient motivation is a key determinant of postoperative satisfaction. Internally motivated patients, driven by personal goals, tend to achieve long‐term satisfaction [8, 24]. Externally motivated individuals, seeking social validation or professional advantage, are more prone to dissatisfaction and depressive symptoms [8]. Motivations rooted in trauma, illness, or systemic disease may also influence outcomes, emphasizing the importance of a trauma‐informed, patient‐centered approach [25, 26]. Understanding patient motivation is critical for both surgical candidacy and postoperative adaptation [26]. Motivations driven by empowerment and emotional healing generally predict positive outcomes, whereas those rooted in avoidance or compulsive appearance monitoring may indicate underlying psychopathology [11]. In such cases, surgery alone is unlikely to meet emotional needs, and psychological intervention should be prioritized [27].

Surgeons have an ethical responsibility to assess both physical and psychological readiness for hair restoration. They must provide clear, evidence‐based information, correct misconceptions, and, when necessary, delay or decline surgery to protect patient well‐being [14, 15]. This includes recognizing and mitigating risks related to unrealistic expectations, mood disorders, or BDD. When motivations appear ambiguous or suggest BDD, delaying surgery and recommending evaluation is a responsible and necessary step [15]. During the preoperative evaluation, physicians should carefully review the patient's psychiatric and pharmacologic history. This includes inquiring about the use of antidepressants, including indications and duration of therapy, as these medications are common and may signal underlying mood disorders. Attention‐deficit/hyperactivity disorder (ADHD) medications may also provide clues to obsessive‐compulsive tendencies. Patients with a history of bipolar disorder or schizophrenia, even if well controlled, warrant consultation with their treating psychiatrist before considering surgery to ensure safety and appropriate management. Some physicians may also incorporate brief personality assessments during the initial consultation. These can help identify traits such as perfectionism, compulsivity, or externally driven motivation that may influence surgical expectations and postoperative satisfaction. While not a substitute for formal psychiatric evaluation, such tools can inform counseling and guide the need for referral. Screening tools such as the BDDQ, PHQ‐9, and GAD‐7 are brief, validated instruments that may help flag patients who can benefit from additional support [16]. Table 2 provides practical guidance for integrating psychological screening into hair transplantation evaluation, helping clinicians identify high‐risk patients and optimize outcomes. Clinics can additionally enhance care by forming partnerships with mental health providers experienced in appearance‐related distress [28]. Such collaboration does not require full psychiatric infrastructure—only a responsive model that prioritizes communication, satisfaction, and mental well‐being.

**TABLE 2:** Practical recommendations for incorporating psychological evaluation in hair transplantation.

Step	Key actions	Tools/Considerations	Clinical pearls
1. Patient history & motivation	Assess hair loss history, treatment attempts, and expectations for surgery. Explore motivation (cosmetic vs. psychosocial)	Structured interview; standardized alopecia history form	Watch for unrealistic goals (e.g., “perfect hairline,” “life will be completely different”)
2. Preoperative counseling	Discuss procedure limits, timelines for results, and need for multiple sessions. Emphasize realistic outcomes	Before/after photos, written materials, counseling session	Set expectations early
3. Psychological screening	Screen for depression, anxiety, body dysmorphic disorder, and social impairment	Body Dysmorphic Disorder Questionnaire (BDDQ), Beck Depression Inventory (BDI), Hospital Anxiety and Depression Scale (HADS)	Positive screens → consider psych referral before proceeding
4. Risk stratification	Identify high‐risk patients (severe psychiatric comorbidity, unrealistic expectations, untreated BDD)	Risk checklist; red flag criteria	Delay or defer surgery if untreated BDD or severe psychiatric illness is suspected
5. Multidisciplinary input	Refer to psychiatry/psychology as needed; collaborate with primary care if mental health issues present	Referral networks, integrated care models	Team approach improves both surgical and mental health outcomes
6. Postoperative support	Monitor psychological adaptation and satisfaction post‐surgery	Follow‐up visits; patient‐reported outcome measures (PROMs)	Early intervention if depressive or anxious symptoms worsen

Patients are generally considered good surgical candidates when they demonstrate (1) stable hair loss patterns, (2) realistic and clearly articulated goals, (3) absence of untreated major psychiatric illness, and (4) willingness to accept limitations of the procedure. Conversely, referral to a mental health professional is indicated when patients screen positive for BDD, present with severe depression or anxiety, exhibit rigid or unrealistic expectations (“this surgery will fix my life”), or display hostility, mistrust, or impaired insight during consultation.

A holistic care model integrating dermatologic, surgical, and psychological expertise is increasingly recommended [13]. Preoperative counseling can clarify motivations, align expectations, and identify vulnerabilities. Postoperative support, including brief psychological interventions or cognitive‐behavioral therapy (CBT), can help patients manage distress during recovery [1].

## 5 Conclusions

Hair transplantation is a multifaceted intervention in which psychological considerations are as critical as technical expertise. Optimal outcomes rely not only on surgical skill but also on the surgeon's ability to identify psychological risks, screen for conditions such as BDD, support realistic motivations, and manage patient expectations with clarity and empathy. Integrating mental health assessment and collaboration with mental health professionals into routine preoperative care is essential for ensuring durable, patient‐centered, and meaningful results as the field continues to evolve.

## Author Contributions

All authors contributed to the ideation and creation of this manuscript.

## Ethics Statement

The authors have nothing to report.

## Consent

The authors have nothing to report.

## Conflicts of Interest

The authors declare no conflicts of interest.

## Data Availability

The data that support the findings of this study are available from the corresponding author upon reasonable request.
